# Loss of AMBRA1 activates MAPK and angiogenesis signaling pathways in melanoma cells

**DOI:** 10.1002/2211-5463.70281

**Published:** 2026-06-02

**Authors:** Milad Ibrahim, Marco Corazzari, Iman Osman, Jane Armstrong, Noel Carter

**Affiliations:** ^1^ Faculty of Health Sciences and Wellbeing University of Sunderland UK; ^2^ The Ronald O. Perelman Department of Dermatology New York University School of Medicine New York NY USA; ^3^ Department of Health Sciences and Translational Center for Autoimmune and Allergic Disease (CAAD) University of Piemonte Orientale Novara Italy

**Keywords:** AMBRA1, angiogenesis, melanoma, tumor suppressor gene

## Abstract

The protein activating molecule in Beclin1‐regulated autophagy1 (AMBRA1), discovered in 2007, is crucial for autophagy and plays roles in nervous system development, cell survival, and proliferation. Here, we investigated AMBRA1's involvement in various cellular processes using a systems‐based “omics” approach, focusing on melanoma.

Transcriptomic analysis of *AMBRA1* overexpression or knock‐down was shown to result in significant dysregulation of several transcripts. We identified several novel roles for AMBRA1 in a range of cellular pathways including cancer signaling pathways such as MAPK, angiogenesis, tissue growth factor signaling, axon guidance, and Wnt signaling. Furthermore, using yeast two‐hybrid assays, we identified novel binding partners which provide evidence of new roles for AMBRA1 in different cellular processes. Ultimately, we conclude that AMBRA1 loss upregulates metastatic genes/proteins highlighting AMBRA1 as a tumor suppressor gene in melanoma.

## Graphical abstract

Loss of AMBRA1 in melanoma cells activates multiple oncogenic pathways associated with tumor progression. Transcriptomic and protein network analyses revealed that AMBRA1 depletion enhances MAPK/ERK signaling, angiogenesis, TGF‐β/EMT signaling, and Wnt/axon guidance pathways. These molecular changes promote melanoma cell proliferation, migration, invasion, angiogenesis, and metastatic potential, supporting a tumor suppressor role for AMBRA1 in melanoma. Created in BioRender. Ibrahim, M. (2026) https://BioRender.com/544gkl6

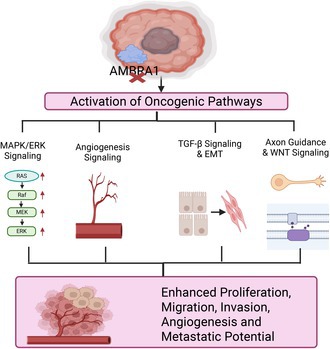

**Abbreviations**
FAK1: Focal adhesion kinase 1
AMBRA1: Activating molecule in Beclin1‐regulated autophagy1
BGal: β‐galactosidase
Depmap: The cancer dependency map
ECM: Extracellular matrix
EMT: Epidermal to mesenchymal transition
MAPK: Mitogen‐Activated Protein Kinase
PCA: principal component analysis
SKCM: Skin Cutaneous Melanoma
TCGA: The Cancer Genome Atlas
Y2H: Yeast‐two hybrid

Melanoma accounts for 75% of skin cancer deaths and remains one of the most therapy‐resistant and aggressive cancers. Survival rates for 5 years after being diagnosed with melanoma are highly dependent on the stage of the disease. If not diagnosed and treated early, melanoma can become highly metastatic. Globally, cases of melanoma are predicted to rise by approximately 50% by 2040 [1, 2]. Considerable improvement in melanoma treatment has been achieved using immunotherapy, targeted therapies, and combination therapies. Despite advancements in melanoma treatment, metastatic disease response remains around 50% [3], underscoring the importance of developing personalized therapies tailored to the molecular features of tumors to improve clinical outcomes [4, 5].

Activating molecule in BECN1‐regulated autophagy 1 (AMBRA1) is a scaffold protein that plays a central role in regulating a range of cellular processes. Its primary identified function is the regulation of autophagy and the development of the nervous system [6]. Advances in the understanding of this protein have shown that it is involved in key physiological events such as metabolism, apoptosis, and cell proliferation. It is also an important regulator of embryonic development [7]. *AMBRA1* has been shown to have a tumor suppressor role in some cancers and a pro‐tumorigenic role in others. For example, AMBRA1 loss in the tumor microenvironment is associated with melanoma metastasis [8, 9], and its loss in the tumor can promote melanoma invasion in a mouse model [10]. The role of *AMBRA1* as a tumor suppressor is supported by its role in associating with PP2CA to dephosphorylate and degrade c‐MYC, leading to decreased cell proliferation [11] and its role in facilitating the degradation of Cyclin D, therefore preventing the overactivation of CDK4/6, which leads to increased cell proliferation [12, 13]. Conversely, studies suggest that higher levels of AMBRA1 have been associated with increased resistance of breast cancer to epirubicin [14] as well as tumorigenesis in breast [15] and gastric cancers [16]. It is also reported to desensitize human prostate cancer cells to cisplatin [17].

In addition to the established roles of AMBRA1 in autophagy and cell proliferation, AMBRA1 also functions in a range of ubiquitin‐mediated mechanisms to regulate protein stability and activity, and as a regulator of gene transcription and protein translation [18, 19]. These functions are underpinned by the ability of AMBRA1 to interact with a wide range of partners, facilitating its role in varied physiological and pathological processes. Despite recent advances, the mechanisms by which AMBRA1 loss promotes tumorigenesis in melanoma are not fully understood. In this study, we analyzed publicly available human melanoma tumor sample DNA, microarray, and RNA‐Seq datasets to assess *AMBRA1* mutations and the levels of its expression in primary and metastatic melanoma. We also performed transcriptional analysis on *AMBRA1* overexpression and knockdown in A375 melanoma cell lines. Finally, we identified putative AMBRA1 protein binding partners. This study proposes novel roles for *AMBRA1* in a range of cellular processes suggestive of a more complex biology.

## Materials and methods

### Bioinformatic analysis

GEO2R tool was used to conduct differential expression analysis on publicly available melanoma datasets. GSE15605 [20] was used to compare AMBRA1 levels in primary, adjacent normal, and metastatic melanoma and GSE59455 [21] was used to compare AMBRA1 levels in primary and metastatic disease. Two TCGA melanoma datasets were analyzed using cbioportal [22]: Skin cutaneous melanoma (TCGA, PanCancer Atlas, UID: 10428) and skin cutaneous melanoma (TCGA, Firehose Legacy, UID: 10490). Normalized counts were downloaded and expression was compared using a Wilcox test. Gepia2 [23] was used for survival analysis of TCGA SKCM which uses log rank test to compare survival. cbioportal [22] was used for mutation analysis of TCGA SKCM (accessed June 2025). Depmap portal (DepMap, Broad (2025). DepMap Public 25Q3. Dataset) was analyzed and accessed via https://depmap.org/portal.

### Cell culture

A375 (RRID:CVCL_0132), MeWo (RRID:CVCL_0445), and SKMEL28 (RRID:CVCL_0526) melanoma cell lines were obtained and authenticated by ATCC using Short Tandem Repeat (STR) profiling and routinely by morphological growth and immunoblot analysis [24, 25, 26]. Cells were cultured and routinely passaged to be maintained in the exponential phase in either high or low Dulbecco's modified eagle medium (DMEM) (Bio‐sera). The media were supplemented with 10% fetal bovine serum (Gibco), 100 μg/mL primocin (Invivogen), and 300 μg/mL L‐glutamine (Lab‐tech). Cells were grown at 37 °C in a 5% CO_2_ humidified atmosphere.

Transfection maintenance was performed by adding antibiotics to the media. The overexpression strains were maintained using G418 at a concentration of 2 μg mL^−1^ and the knock‐down cell lines were maintained using puromycin at a concentration of 2 μg mL^−1^. Cell lines. All experiments were performed in mycoplasma‐free cell lines.

### Lentiviral production and infection

Lentiviral particles were generated by co‐transfecting 293 T packaging cells with a total of 10 μg of shRNA‐expressing pLKO vectors targeting *AMBRA1* (Merck: Predesigned shRNA: TRCN0000168652: GCATGTGGACTCTTAACTGTA, TRCN0000425683: AGCCTCTCTTCGTCGTCTTAC, and TRCN0000167886: CCCACTTTCTCCTAGTAACAT) or non‐targeting control (SHC016‐1EA), 2.5 μg of a vesicular stomatitis virus G (VSV‐G) envelope expression plasmid, and the psPAX2 packaging plasmid (which encodes the *gag*, *pol*, and *env* genes). Transfections were carried out using the calcium phosphate precipitation method [27]. At 48 h post‐transfection, the culture supernatants containing lentiviral particles were harvested and filtered through a 0.45‐μm pore filter to remove cellular debris. To enhance transduction efficiency, polybrene was added to a final concentration of 4 μg mL^−1^. The viral supernatant was then used to infect A375 cells. Following selection, the knockdown efficiency of each shRNA construct was evaluated at 48 h by qPCR, and the most effective shRNA (shRNA: TRCN0000168652: GCATGTGGACTCTTAACTGTA) was selected for use in subsequent experimental assays [28].

### Retroviral production and infection

Retroviral particles were produced by co‐transfecting 293 gp/bsr packaging cells with 15 μg of retroviral vectors encoding AMBRA1 (overexpression construct) or β‐Galactosidase as a control [29] and 5 μg of a vesicular stomatitis virus G (VSV‐G) envelope protein expression plasmid. Transfection was carried out using the calcium phosphate precipitation method. Forty‐eight hours post‐transfection, culture supernatants containing retroviral particles were collected, filtered, and supplemented with 4 μg mL^−1^ polybrene to enhance infection efficiency. A375 cells were then transduced by incubating them with the viral supernatant for 6–8 h. Overexpression of AMBRA1 or *β*‐Galactosidase in infected A375 cells was subsequently confirmed by quantitative PCR (qPCR).

### Incucyte live cell growth assay

Two thousand cells per well were seeded in 96‐well plates. Nine wells were used as replicates for each cell line condition. Live cells were imaged using the incucyte system (Essen BioScience), taking four images per well every 2 h for 5 days after the cells were seeded. incucyte software was used to segment and process object count (cells per image) and represent it as the average number of cells per image over time. Data are presented as mean+/− standard deviation. *P*‐values were calculated using an independent, two‐sided *t*‐test by comparing the non‐linear fit of exponential growth of each cell line against its control, and significance was calculated as *P* < 0.05.

### Western blot analysis

Cells were seeded at a concentration of 5 × 10^5^ to a 6‐well plate and grown at 37 °C and maintained in the exponential phase. Cells were then washed twice with PBS and harvested with 200 μL 1× cell lysis buffer (Abcam, Cambridge, UK) and sonicated for 3 × 10 seconds. Lysates were stored at −80 °C and used for downstream analysis. Protein quantification was performed using BSA as a standard and Bradford reagent according to the manufacturer's instructions. Approximately 25 μg of protein was resolved on a 12% stain‐free TGX polyacrylamide gel (Bio‐Rad, Watford, UK) electrophoresis. A stain‐free fluorescent image of the gel was captured before gels were transferred to polyvinylidene difluoride (PVDF) using the transblot turbo system (Bio‐Rad). Membranes were blocked in Immobilon® Block‐Chemiluminescent Blocker (CB) (Merck, Feltham, UK) for 1 h at room temperature. Membranes were then incubated with primary antibodies overnight at 4 °C with shaking, washed 3 × 10 minutes with TBST and incubated with secondary antibodies for 1 h at room temperature followed by another 3 × 10 minutes wash, membranes were then incubated with 2 mL freshly prepared (1 : 1) mixture of peroxide reagent and luminol/enhancer reagent supplied from Bio‐Rad for chemiluminescent detection of HRP activity of the conjugated secondary antibody. The membranes were then analyzed by the ChemiDoc™ Imaging System (Bio‐Rad, UK) for protein band detection. Antibodies used: Rabbit anti human AMBRA1 antibody (00013214; Covalab, Cambridge, UK), Chicken anti‐beta‐Galactosidase (GW20071F; Sigma, Cambridge, UK), Mouse monoclonal to FLT1 (VEGF receptor 1) (ab9540; Abcam), Rabbit monoclonal to Wnt5a‐C‐terminal (ab235966; Abcam), Goat anti‐chicken IGY (ab97135; Abcam), Goat anti‐rabbit IgG (AI‐1000‐1.5; Vector labs), and Goat anti‐mouse IgG (AI‐9200‐1.5; Vector labs, North Yorkshire, UK). Quantitative analysis was performed using imagelab version 4.0 (Bio‐Rad). Pixel intensities of western bands were quantified by creating identical volume boxes and using a separate background volume to perform background subtraction. Total protein loading per lane was quantified by generating the pixel intensities of the protein loading from the stain free gel image as outlined above. The relative pixel intensities per lane were normalized to a nominal total protein lane set as a nominal value of 1. The normalized relative pixel intensities of the western bands were calculated as their band intensities divided by the normalized protein loading value (Fig. S2). The use of stain‐free gels for protein normalization has been shown to have advantages over performing a separate loading control [30, 31].

### RNA extraction and quality analysis

Total RNA was extracted from each cell line using Trizol reagent (Thermo‐Fisher, Leicestershire, UK, Cat. 15 596 026) according to the manufacturer's instructions and the precipitated RNA was re‐suspended in 200 μL 1 × RNA secure reagent (Ambion, Leicestershire, UK). RNA quality analysis was performed using Experion™ RNA StdSens and HighSens Analysis Kits from Bio‐Rad following instructions from the manufacturer.

### Gene expression analysis by microarray

The analysis was performed by Source Bio‐Science. Three biological replicates from each cell line at a concentration of 120 ng μL^−1^ were hybridized on GeneChip™ Human Gene 2.0 ST. The microarray data were normalized and analyzed using transcriptome analysis suite (TAC version 4.0.1) (Thermo‐fisher). Ebayes ANOVA method was used, and the analysis was set to a *P*‐value < 0.05 and FDR < 0.09. Functional analyses were performed using Gene Ontology (GO) resource [32, 33] and STRING protein–protein interaction network [34, 35].

### Yeast‐two hybrid protein–protein interaction

Yeast strains were maintained by streaking at least once every 4 weeks on fresh YPDA plates, incubated at 30 °C for 3–5 days then stored at 4 °C. Only fresh grown colonies were used for transformations. Plates were prepared by adding each media pouch content to 500 mL deionized water then autoclaving, left to cool to 50 °C then X‐α‐gal and/or antibiotics were added before pouring the plates. Yeast transformation, positive and negative control experiments were performed as stated by yeast‐two hybrid system manufacturer (Clontech Laboratories, London, UK). All isolated plasmids were sequenced using a t7 promoter at Source Bio‐science limited.

Yeast transformation, positive and negative control experiments were performed as stated by Yeast‐Two Hybrid System manufacturer (Clontech Laboratories). A positive control mating was performed by mating Y2H gold strain fused with murine p53 and Y187 yeast strain fused with SV40 large T‐antigen. A negative control experiment was performed by mating Y2h gold yeast stain fused with lamin and Y187 yeast strain fused with SV40 large T‐antigen.

### cDNA synthesis and PCR amplification

PPP2CA and AMBRA1 cDNAs were prepared using oligo primers by PCR using RNA extracted from U‐937 (ATCC® CRL‐1593.2™) and A‐375 (ATCC® CRL‐1619™) cell lines. An ORF clone with AMBRA1 sequence was purchased to be used as a PCR template. PCR reactions were performed on a Bio‐Rad T100 thermal cycler. PCR reactions were performed using 10 × Immomix master mixes (Bio‐Rad), Q5 polymerase (Bio‐labs), and proof‐reading polymerase was later used (Iproof Bio‐Rad).

Primers:

PPP2CA‐Forward 5′‐ CGC GAA TTC ATG GAC GAG AAG GTG TTC ACC ‐3′.

PPP2CA‐Reverse 5′‐ CGC GGA TCC TTA CAG GAA GTA GTC TGG GGT ACG ‐3′.

AMBRA1‐Forward 5′‐ AGG AGG ACC TGC ATA TGA AGG TTG TCC CAG AAA AGA ATG CC ‐3′.

AMBRA1‐Reverse 5′ GCC TCC ATG GCC ATA CTA CCT GTT CCG TGG TTC TCC C‐3′.

### DNA digestion, ligation, plasmid extraction from *E. coli* and transformation into yeast cells

Plasmids were transformed to competent *E. coli* (DH5‐α strain, Invitrogen 18 258 012) according to the manufacturer's protocol using the appropriate antibiotic for selection. Plasmids were transformed into the yeast strains according to the manufacturer's specifications (Clontech). Plasmids were extracted from *E. coli* using the plasmid plus midi kit (QIAGEN technologies) according to the manufacturer's instructions. All of the PCR products and plasmids were digested by using 200 ng DNA to 1 μL of the restriction enzymes (*BamH* I, *EcoR* I, or *Nde* I, Thermo Scientific). All ligation reactions were made at a vector‐to‐insert ratio of 1 : 5; the appropriate ligase was added to the reaction mixture and incubated overnight at room temperature.

### Statistical analyses

Statistical analyses were performed with Microsoft Excel, graphpad Prism (version 10.2.0, RRID:SCR_002798), or *R* statistical software (RRID:SCR_001905 version 4.3.1).

## Results

### AMBRA1 expression in melanoma

**Fig. 1:**
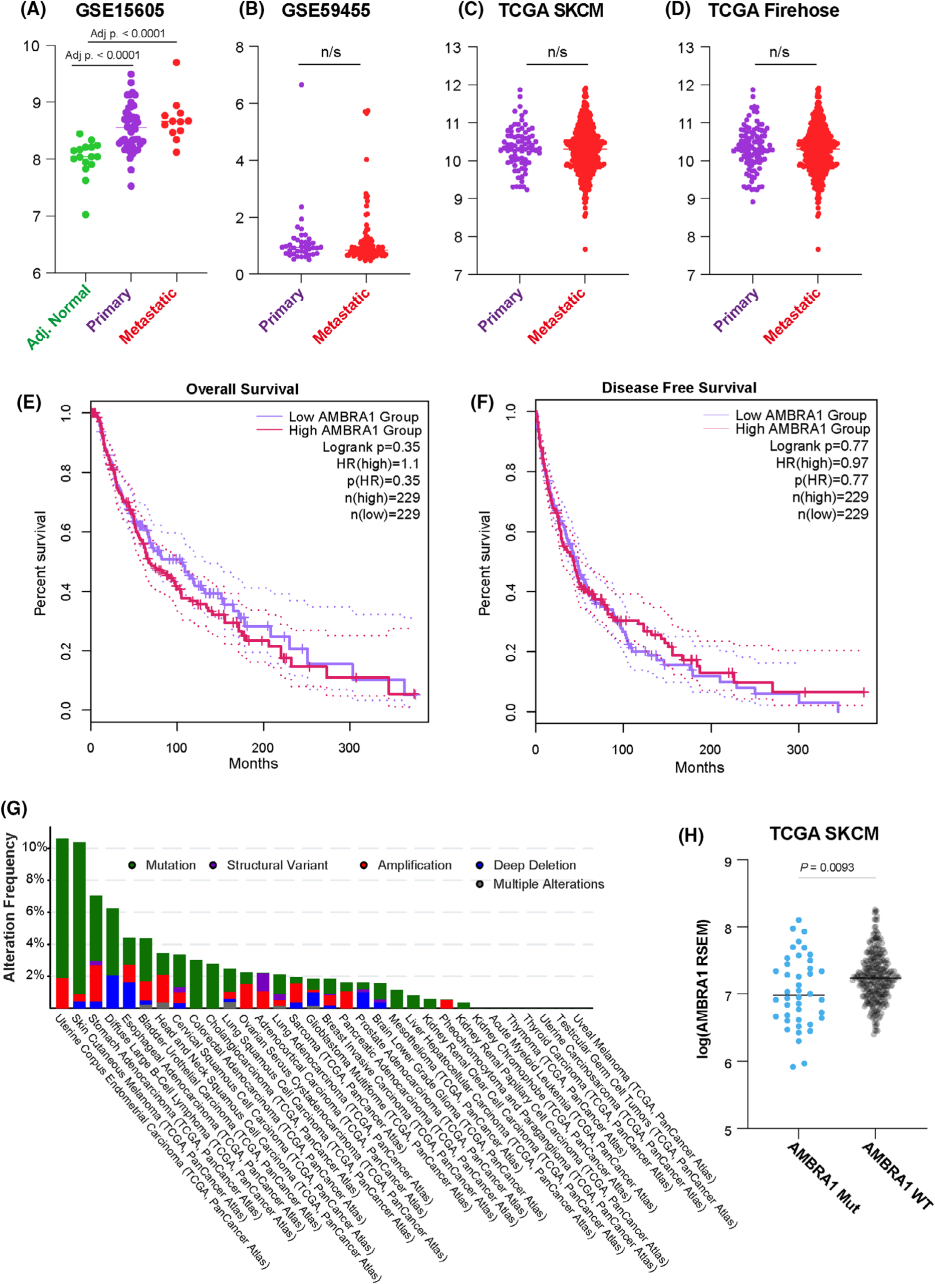
AMBRA1 expression and mutation in melanoma. (A–D) Dot plot showing the expression of AMBRA1 in four different melanoma datasets: (A) GSE15605, adjacent normal skin (*n* = 16), primary (*n* = 46), and metastatic (*n* = 12) melanomas; (B) GSE59455, primary (*n* = 39), metastatic (*n* = 102) melanomas; (C) TCGA SKCM, primary (*n* = 109), metastatic (*n* = 371) melanomas; (D) TCGA Firehose, primary (*n* = 81), metastatic (*n* = 367) melanomas. *P*‐values are calculated by a two‐sided *t*‐test. (E, F) Kaplan–Meier graphs showing overall (E) and disease‐free (F) survival in high and low AMBRA1 expression in melanoma (*n* = 458). Dotted lines represent the 95% confidence intervals (CI). *P*‐values are calculated by log‐rank test. (G) Bar plot showing AMBRA1 mutation percentage in all cancer types. (H) Dot plot showing the expression of AMBRA1 in AMBRA1 mutant (*n* = 46) or wild‐type (*n* = 400) melanoma patients in TCGA SKCM. *P*‐values are calculated by a two‐sided *t*‐test.

It is proposed that AMBRA1 loss drives melanoma development and metastasis through promotion of cell proliferation followed by activation of diverse pathways involved in tumor progression. Melanoma cell lines and tumors exhibit a range of *AMBRA1* expression [10], but it is not known whether expression levels change during progression to metastatic disease. Here, we performed a bioinformatic analysis on publicly available datasets to investigate the levels of *AMBRA1* in melanoma. First, we compared *AMBRA1* expression levels in GSE15605 [20] which included 46 primary, 16 adjacent normal, and 12 metastatic tissue isolated from melanoma patients. *AMBRA1* expression was significantly decreased in adjacent skin compared to primary (*P* < 0.001) and metastatic melanoma (*P* < 0.001) (Fig. 1A). Next, we tested AMBRA1 expression levels in three different datasets: GSE59455 [21] which included 39 primary and 102 metastatic samples, Skin Cutaneous Melanoma (SKCM, TCGA, PanCancer Atlas, UID: 10428) which included 109 primary and 371 metastatic samples, and Skin Cutaneous Melanoma (TCGA, Firehose Legacy, UID: 10490) which included 81 primary and 367 metastatic samples, and found no difference between *AMBRA1* levels in primary and metastatic melanoma (Fig. 1B–D).

*AMBRA1* expression levels did not correlate with overall survival (Fig. 1E) or disease‐free survival (Fig. 1F) in the Cancer Genome Atlas Program (TCGA) Skin Cutaneous Melanoma (TCGA, PanCancer Atlas, UID: 10428). *AMBRA1* mutations were reported in 10% of SKCM, and melanoma is the second type of cancer that was enriched for *AMBRA1* mutations after Uterine Corpus Endometrial Carcinoma (Fig. 1G). *AMBRA1* mutations were predominantly missense (35 events), followed by truncating (9 events) and a single splice‐site mutation. These alterations were distributed across the *AMBRA1* gene and did not cluster within a specific functional domain (Fig. S1). These loss‐of‐function mutations resulted in significantly decreased *AMBRA1* mRNA levels (*P* = 0.0093) (Fig. 1H). Together, these results indicate that a subset of melanoma tumors favor AMBRA1 loss.

### Effect of AMBRA1 knockdown on melanoma cell growth

**Fig. 2:**
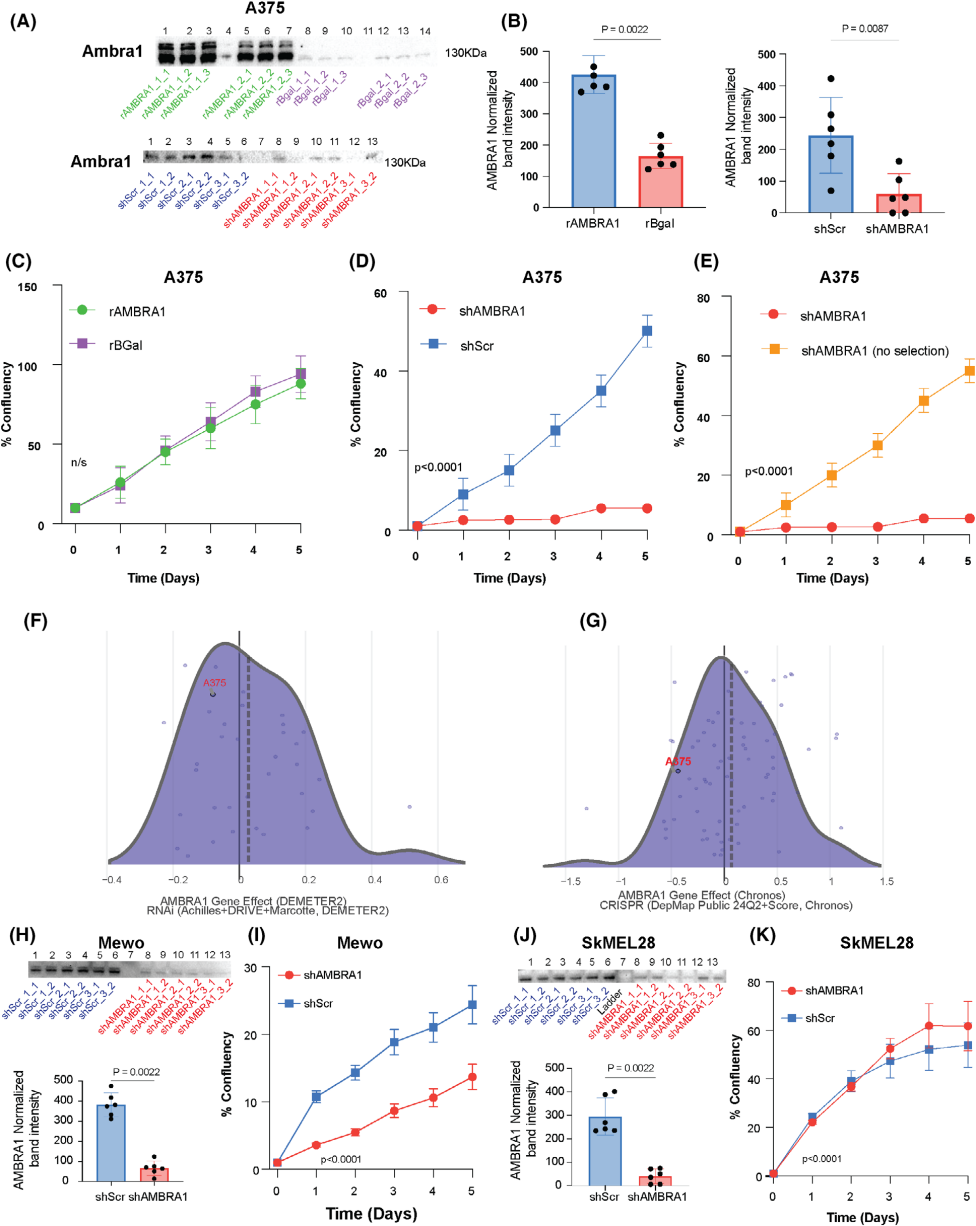
AMBRA1 loss decreased A375 melanoma cell proliferation (A) Western blot analysis showing AMBRA1 overexpression (Top), lanes 1–3 and 5–7 are three technical replicates of two biological rAMBRA1 replicates and lanes 8–10 and 12–14 are three technical replicates of two biological rBgal replicates or Knockdown (bottom) in A375 melanoma cell lines, lanes 1–6 are two technical replicates of three biological shScramble replicates and lanes 8–13 are two technical replicates of three biological shAMBRA1 replicates. (B) Bar graphs showing western blot data normalized to total protein in rAMBRA1 versus rBgal (Left) or shScramble versus shAMBRA1 (Right). Bars show mean values ± SD. *P*‐values are calculated by a Wilcox test. (C–E) Growth curves of A375 cell lines expressing (C) rAMBRA1 and rBgal, (D) shAMBRA1 and shScramble or (E) shAMBBRA1 and shAMBRA1 with no selection. Data points indicate mean ± SD (*n* = 9). *P*‐values are calculated using a two‐tailed unpaired *t*‐test comparing the non‐linear fit of exponential growth. (F, G) DepMap Chronos score plot showing the effect of AMBRA1. (F) Knockdown by RNAi or (G) knockout by CRISPR in melanoma cell lines. Each melanoma cell line is represented by a single dot on the graph. A cell line with a positive score means that the growth of these cells is not affected by AMBRA1 depletion. Conversely, a negative score indicates decreased proliferation upon AMBRA1 depletion. The solid line represents zero and the dotted line represents the median of score across cell lines. The position of the cell line A375 is highlighted in both datasets. (H) (Top) Western blot analysis showing AMBRA1 knockdown in Mewo melanoma cell lines, lanes 1–6 are two technical replicates of three biological shScramble replicates and lanes 8–13 are two technical replicates of three biological shAMBRA1 replicates. (Bottom) Bar graphs showing western blot data normalized to total protein in shScramble versus shAMBRA1. Bars show mean values ± SD. *P*‐values are calculated by a Wilcox test. (I) Growth curves of Mewo cell lines expressing shAMBRA1 and shScramble. *P*‐values are calculated using a two‐tailed unpaired *t*‐test comparing the non‐linear fit of exponential growth. (J) (Top) Western blot analysis showing AMBRA1 knockdown in Mewo melanoma cell lines, lanes 1–6 are two technical of three biological shScramble replicates and lanes 8–13 are two technical of three biological shAMBRA1 replicates. Bars show mean values ± SD. *P*‐values are calculated by a Wilcox test. (Bottom) Bar graphs showing western blot data normalized to total protein in shScramble versus shAMBRA1. (K) Growth curves of Mewo cell lines expressing shAMBRA1 and shScramble. *P*‐values are calculated using a two‐tailed unpaired *t*‐test comparing the non‐linear fit of exponential growth.

The mechanisms by which AMBRA1 loss regulates increased cell proliferation are well understood [11, 12]; however, AMBRA1 depletion can promote the growth of certain cell types [16, 36] implying an AMBRA1 dependency in some contexts. Melanoma cell lines exhibit a range of AMBRA1 protein abundance [10], suggesting AMBRA1 dependency may be cell line specific. To test the effect of *AMBRA1* expression levels on cell growth in a model system, we utilized A375 melanoma cells overexpressing AMBRA1 (rAMBRA1) or A375 AMBRA1 depleted cells (shAMBRA1) (Figs 2A,B and S2A,B) and their respective controls overexpressing β‐galactosidase (rBGal) or a scrambled sequence shRNA control (shScramble). Live cell imaging assays showed that AMBRA1 overexpression did not affect the growth of the melanoma cell line (Fig. 2C). Conversely, AMBRA1 depletion led to a significant decrease in melanoma cell growth over time (Fig. 2D). Of note, after 2 weeks of removing the shAMBRA1 construct selection antibiotic, the cells restored their ability to grow, indicating that the cells favor AMBRA1 expression, which was partially restored after removing selection (Figs 2E and S3A,B). These data suggest that A375 cells in culture are dependent on AMBRA1 expression.

To validate our findings, we navigated the cancer dependency map (Depmap) portal [37] which defines which genes are essential for cancer cell survival. A negative score indicates that a gene is essential, and its loss decreases the proliferation of cancer cells, while a positive score indicates that loss of the gene does not affect cancer cell proliferation. In other words, a cell line with a positive score means that the growth of these cells is not affected by AMBRA1 depletion. Conversely, a negative score indicates decreased proliferation upon AMBRA1 depletion. Depmap analysis confirmed our observation of decreased cell proliferation after *AMBRA1* knockdown in A375 cells using RNAi (Fig. 2F, File S1) and knockout using CRISPR (Fig. 2G, File S2). Furthermore, Mewo, SKMEL3, COLO741, A2058, SKMEL2, and LOXIMVI cell lines showed negative *AMBRA1* dependency scores, consistent with a dependency on AMBRA1. Conversely, SKMEL5, SKMEL28, WM115, WM1799, SH4, and RVH421 cell lines showed positive AMBRA1 dependency scores, suggesting that loss of AMBRA1 has minimal impact on cell proliferation in these models.

Given the context‐dependent effects of AMBRA1 depletion on melanoma cell proliferation observed in DepMap, we sought to experimentally validate these findings. To this end, we performed AMBRA1 knockdown in two additional melanoma cell lines: Mewo, which exhibits a negative Chronos score (predicting reduced proliferation upon AMBRA1 loss), and SKMEL28, which exhibits a positive Chronos score (predicting no dependency on AMBRA1). Immunostaining confirmed efficient AMBRA1 depletion in both models (Figs 2H, J and S2C,D). Consistent with DepMap predictions, AMBRA1 knockdown significantly reduced proliferation in MeWo cells (Fig. 2I), while not decreasing proliferation in SK‐MEL‐28 cells (Fig. 2K). Collectively these results suggest that the role of *AMBRA1* in melanoma cell proliferation can be cell and context dependent.

### AMBRA1 plays a role in regulating hallmark melanoma pathways

**Fig. 3:**
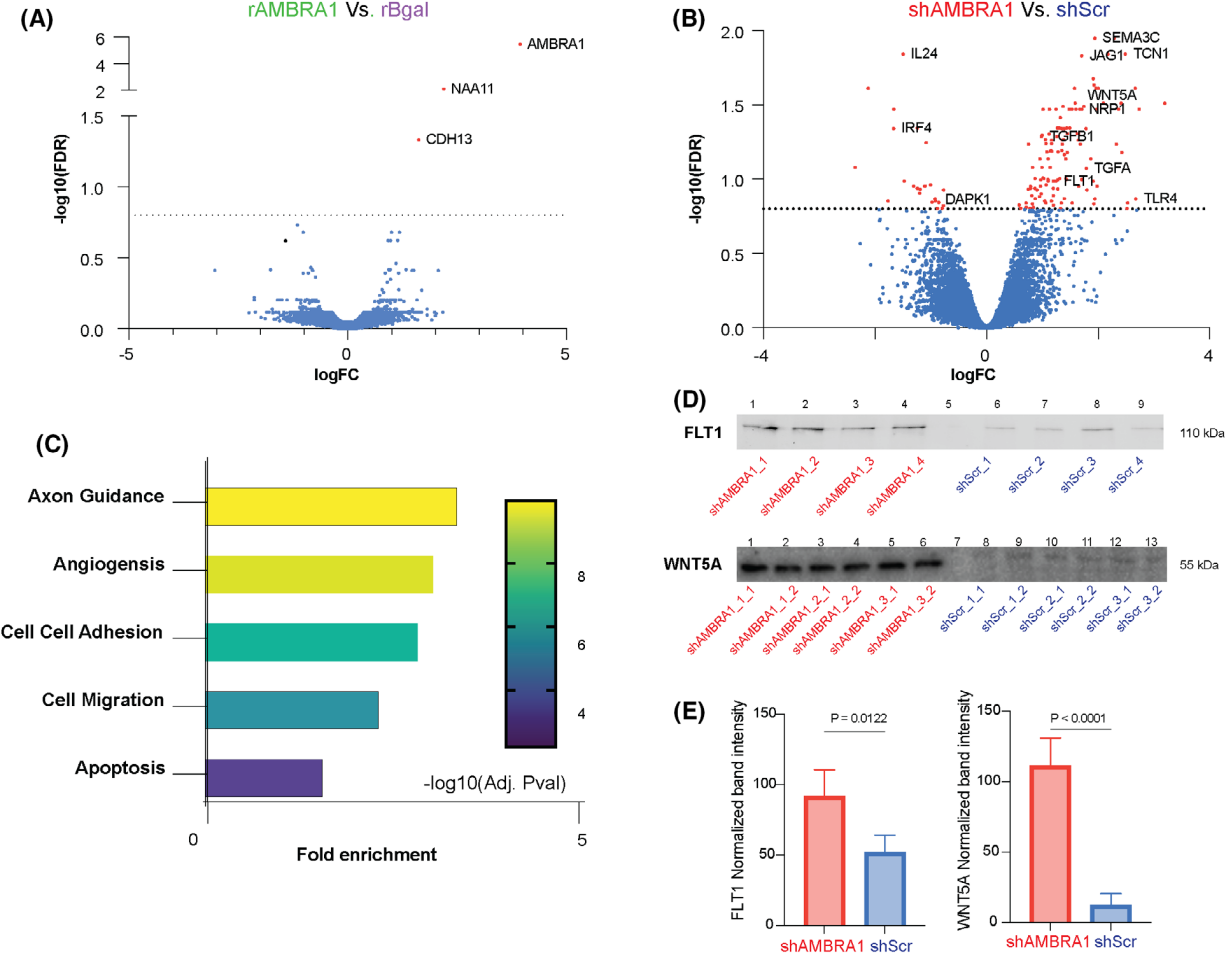
AMBRA1 regulate hallmark cellular processes on the transcriptional level (A,B) Volcano plots showing differentially expressed genes between rAMBRA1 and rBgal (A) or shAMBRA1 and shScramble (B). (C) GO biological processes analysis of differential expressed genes in shAMBRA1 compared to shScramble. (D, E) Western blot analysis showing FLT1 and Wnt5A expression in A375 melanoma cell lines comparing shAMBRA1 and shScramble. *P*‐values were calculated using a two‐tailed *t*‐test. FLT1 lanes 1–4 are two technical replicates of two shAMBRA1 biological replicates. Lanes 6–9 are two technical replicates of two shScramble biological replicates. WNT5A lanes 1–6 are two technical replicates of three shAMBRA1 biological replicates. Lanes 6–9 are two technical replicates of three shScramble biological replicates. (E) Bar plots showing normalized band intensities of (D). Bar graphs indicate mean ± SD. *P*‐values were calculated with a Wilcox test.

Our findings indicate that AMBRA1 role in regulating cell proliferation is complex. To investigate the molecular pathways that can be regulated by *AMBRA1*, we performed transcriptomic analysis using A375 cells with either the overexpression or knockdown of AMBRA1. 3D principal component analysis (PCA) revealed that shAMBRA1 clustered separately from the other cell lines (Fig. S4). As expected, in rAMBRA1 cell lines, *AMBRA1* expression led to a significant increase in *AMBRA1* transcript levels. Beyond AMBRA1 itself, *NAA11* and *CDH13* were the only additional transcripts significantly upregulated compared to control cells. (Fig. 3A, File S3).

**Fig. 4:**
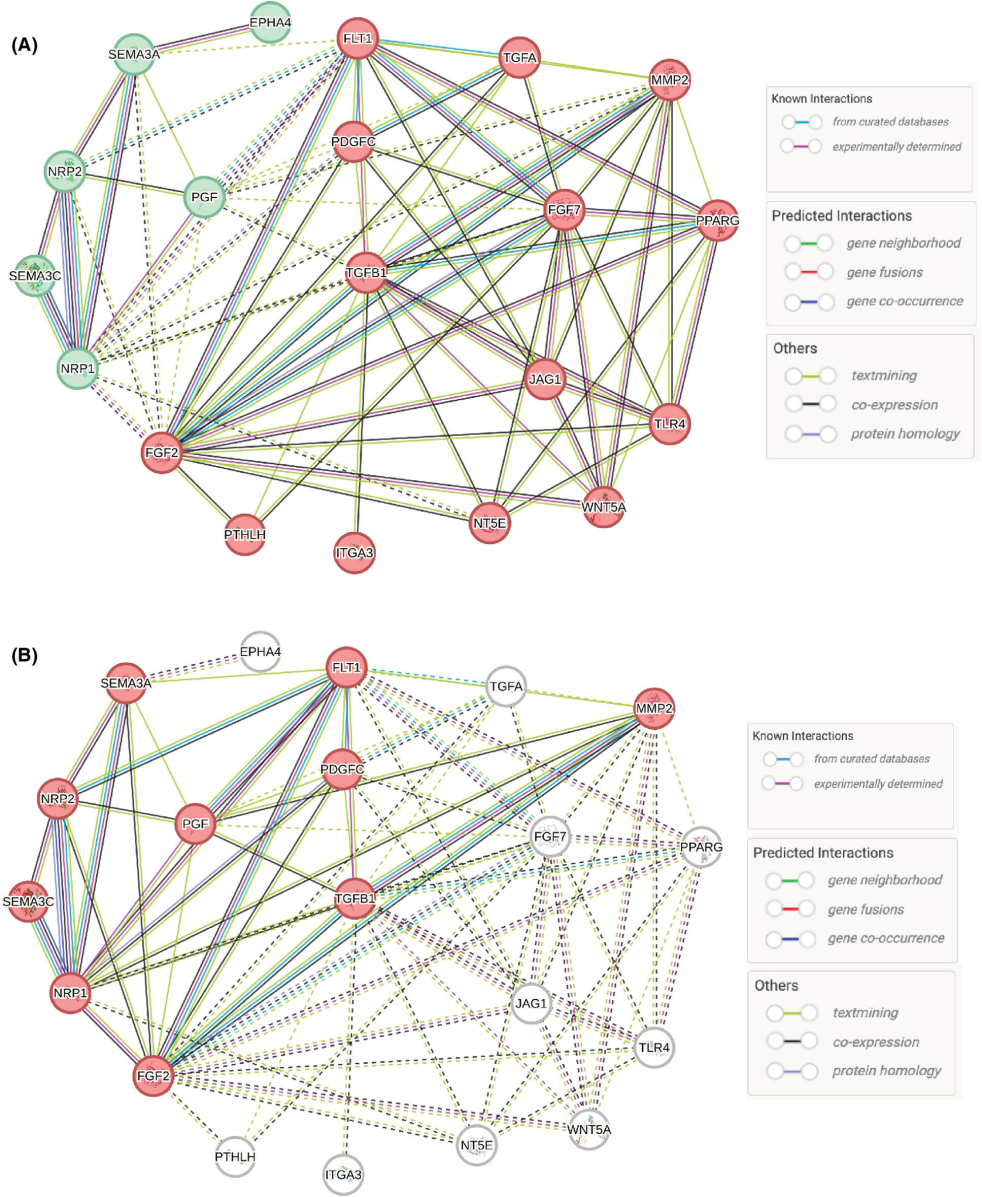
AMBRA1 loss leads to the activation of MAPK and angiogenesis signaling. (A) STRING protein network analysis of overexpressed proteins resulting from AMBRA1 knockdown showing that these proteins form a network of interactions that is involved in MAPK signaling. All the proteins forming the network are shown with the ones enriched in MAPK signaling highlighted in red and connected in solid lines. Each protein is represented as a node and interactions are represented as edges. (B) STRING protein network analysis of overexpressed proteins resulting from AMBRA1 knockdown showing that these proteins form a network of interactions that is involved in angiogenesis. All the proteins forming the network are shown with the ones enriched in angiogenesis highlighted in red and connected in solid lines. Each protein is represented as a node and interactions are represented as edges.

AMBRA1 knockdown resulted in 103 and 11 genes being significantly up‐ and downregulated (*P* < 0.05, FDR < 0.09, −2 > log2fc > 2), respectively. Among differentially expressed genes, *AMBRA1*, *DAPK1*, *IL24*, and *IRF4* were downregulated in shAMBRA1 cells. Conversely, *SEMA3C, JAG1, TCN1, WNT5A, FLT1, NRP1, TGFB1, TGFA*, and *TLR4* were upregulated (Fig. 3B, File S4). These upregulated genes were enriched for axon guidance, angiogenesis, cell–cell adhesion, cell migration, and apoptosis pathways (Fig. 3C). Western blot analysis confirmed the increased expression of FLT1 and WNT5A in A375 cells with *AMBRA1* knockdown (Figs 3D,E, S2E,F). To gain further insights into AMBRA1's role in regulating cell proliferation, we generated protein–protein interaction networks of the transcripts that were upregulated as a result of AMBRA1 loss. STRING analysis showed that AMBRA1 loss leads to the upregulation of genes that form a network of interactions centered around MAPK activity (Figs 4A and S5A) and included *TGFA, TGFB1, FGF2, FGF7, ITGA3*, and *NT5E*. In addition, AMBRA1 loss upregulates *FLT1*, *SEMA3C*, *PGF*, and *PDGFC*, which are key regulators of angiogenesis (Fig. 4B).

Collectively, these data suggest that the functional role of AMBRA1 extends beyond autophagy and cell proliferation regulation, and that AMBRA1 can regulate hallmark cellular pathways like angiogenesis and MAPK activity.

### Novel AMBRA1 binding partners

**Fig. 5:**
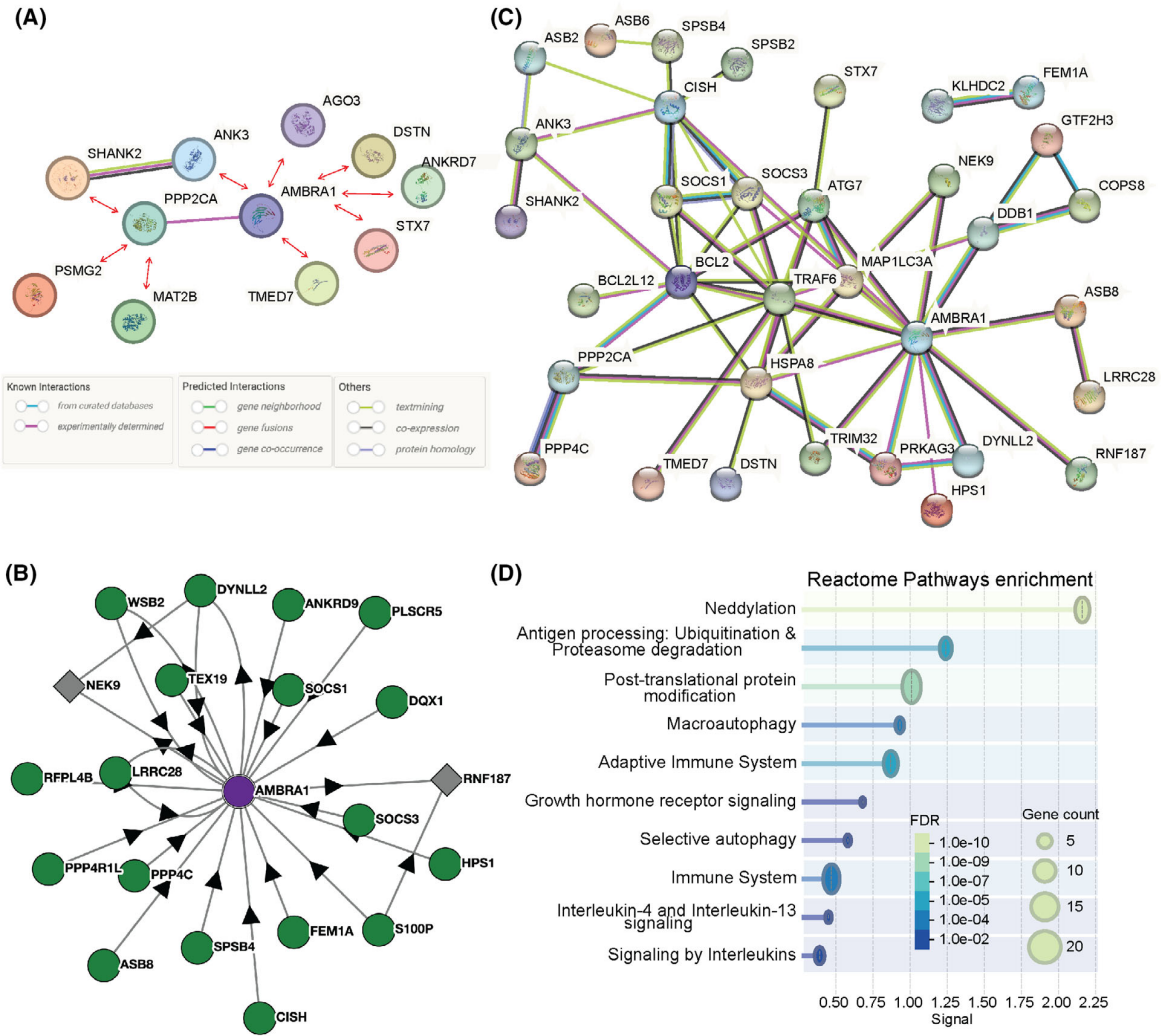
Novel AMBRA1‐binding partners (A) STRING protein interaction network of AMBRA1 and PPP2CA; novel interactions identified in this study are marked with red arrows. (B) Bioplex portal protein–protein interaction database showing AMBRA1 binding partners identified by high‐throughput mass spectrometry. (C) STRING protein interaction network of AMBRA1 binding partners identified in literature, BioPlex, and this study. Each protein is represented as a node and interactions are represented as edges. Disconnected nodes were removed for simplification. (D) Reactome pathway analysis of the protein network was identified in (C).

Since AMBRA1 is a scaffold protein that can interact with a large number of other proteins [6], we performed a yeast‐two hybrid (Y2H) experiment using AMBRA1 as a bait to test for novel protein–protein interactions. We identified seven novel AMBRA1 protein‐binding partners: STX7, TMED7, ANK3, DSTN, AGO3, and ANKRD7. Of note, none of these interactions have been previously reported. We also used PPP2CA as a positive control, as studies suggested that it can bind AMBRA1 [11]. Y2H experiments picked MAT2B, SHANK2, and PSMG2 as PPP2CA interactors but not AMBRA1, possibly due to the limited number of screened positives. Collectively, we report novel AMBRA1 and PPP2CA binding partners that can constitute a larger network of proteins (Fig. 5A).

To explore a more comprehensive AMBRA1 network, we searched reported AMBRA1 binding partners in the interactome BioPlex database (Fig. 5B) [38]. We then combined AMBRA1 binding partners identified in our study with partners previously reported in literature and in BioPlex. This integration resulted in a more complex protein network built around AMBRA1 (Fig. 5C), and this network confirms a role for AMBRA1 in autophagy and ubiquitination and suggests novel roles of AMBRA1 in the immune system and interleukin signaling (Fig. 5D). Of note, transcriptional analysis shows that AMBRA1 loss downregulates IL24 and IRF4 (Fig. 3B), further indicating a role of AMBRA1 in interleukin signaling and the immune system.

## Discussion

Since its discovery in 2007, AMBRA1 research has significantly advanced, with a reported role in regulating several key biological pathways [6, 7, 10, 11]. Specifically, the role of AMBRA1 in melanoma was highlighted in recent years as it was shown that AMBRA1 loss in the epidermis surrounding melanoma is a biomarker indicating worse prognosis [8, 9]. However, in other cancer types, the role of AMBRA1 appears to be dynamic and can vary between one type of cancer and another [15, 17]. The mechanisms by which AMBRA1 can play such roles remain largely unclear. Our study describes novel roles of AMBRA1 that can potentially contribute, at least in part, to its role in melanoma and different cancer types.

Our bioinformatic analysis showed that *AMBRA1* is increased in melanoma tumors compared to the skin adjacent to melanoma; however, *AMBRA1* levels were not different in metastatic compared to primary melanoma in four independent datasets. Moreover, TCGA data analysis suggests that *AMBRA1* mutations that lead to subsequent loss of expression are highly prevalent in melanoma. These data suggest that a subset of tumors could favor AMBRA1 loss.

Our findings align with recent studies that showed AMBRA1 loss can promote melanoma invasion and metastasis [10]. In this study, we report that, in metastatic A375 melanoma cells, the overexpression of *AMBRA1* did not appear to influence the proliferation rate in an environment where nutrition is not limited. However, knockdown of *AMBRA1* resulted in decreased cell proliferation. This result was not expected, as AMBRA1 loss has been shown to promote melanoma growth and invasion [10]. However, analyzing the Depmap portal showed that *AMBRA1* gene knockdown or knockout can have a dual effect on different melanoma cell lines, and it had a negative effect on the proliferation in A375 similar to what we observed in our study. A simple explanation is that the blockage of autophagy by *AMBRA1* knockdown is causing this decreased proliferation under cell culture conditions, as melanoma cells need the autophagy machinery to avoid apoptosis and cell death [39, 40]. We noticed that the cells favored AMBRA1 expression as cell proliferation was restored after 2 weeks of shRNA selection removal.

*AMBRA1* is considered a tumor suppressor in melanoma, loss of which promotes tumor growth and an invasive phenotype through enhanced cell proliferation (by stabilization of c‐MYC and D‐type cyclins), activation of focal adhesion kinase 1 (FAK1) signaling, extracellular matrix (ECM) remodeling, and upregulation of epidermal to mesenchymal transition (EMT) genes [11, 12, 13, 41, 42]. Our study suggests that tumors may favor AMBRA1 loss as it leads to the activation of pathways providing tumors with an advantage of increased proliferation and metastatic potential. We show that *AMBRA1* loss leads to the upregulation of canonical Wnt signaling via *FZD8* overexpression and non‐canonical Wnt signaling via *WNT5A* overexpression and through binding ANK3, a regulator of Wnt signaling [27]. The Wnt family of proteins is involved in the regulation of cell proliferation, cell motility, cell polarity, organogenesis, cell fate, and stem cell renewals [28]. WNT5A is a member of the Wnt family that signals through both the canonical and non‐canonical Wnt pathways but it is most often associated with non‐canonical Wnt signaling [29]. The role of WNT5A in cancer remains under investigation [30], but evidence suggests that it can have a pro‐tumorigenic role in melanoma [31, 32].

We also show that AMBRA1 loss leads to the activation of MAPK signaling and associated pathways. Consistent with AMBRA1 loss conferring metastatic potential, in our model, *AMBRA1* inhibition led to the upregulation of genes involved in tumor cell survival and migration (*FGF, ITGA3*), EMT transition (*TGFB1*), angiogenesis (*PGF*), invasion and metastasis (*NT5E, PDGFC*), including through autocrine/paracrine signaling in the tumor microenvironment [43, 44, 45, 46, 47, 48].

In this study, we have also identified an interaction between AMBRA1 and ANKRD7, an effector of the small RAB GTPases RAB32 and RAB38, which can indicate a possible regulation of the RAS pathway, a hallmark of cell proliferation [49], by AMBRA1. Furthermore, AGO3 was among the proteins identified to interact with AMBRA1. This protein is reported to have a role in stem cell proliferation, regulating gene expression, and it is essential in human embryogenesis [50]. AMBRA1 has also been reported to be implicated in embryogenesis [6]. Therefore, AMBRA1 and AGO3 might regulate one another during these key biological processes.

In addition, our study suggests novel roles of AMBRA1 in regulating multiple cellular pathways. For example, AMBRA1 was shown to be essential for nervous system development during embryogenesis and is highly expressed in various neural tissues [6]. Our study suggests a role for AMBRA1 in regulating axon guidance signaling, which is the process by which neuronal axons grow to reach their targets and establish connections, by altering the expression of key genes in this pathway. Knockdown of AMBRA1 significantly affects axon guidance. Additionally, ANK3, an identified AMBRA1 binding partner, is involved in axon segment initiation [51].

Our study suggests that AMBRA1 functions within a broader protein network that can modulate immune responses. Consistent with this notion, recent studies have shown that AMBRA1 plays a role in shaping tumor–immune interactions. Melanoma secretion of IL‐24 can promote an immune response through increased CD8+ T‐cell activity [52]. We show that AMBRA1 knockdown is associated with a downregulation of *IL24*, suggesting AMBRA loss can negatively impact the tumor immune microenvironment, consistent with the reported association between AMBRA1 loss and reduced tumor infiltration by immune‐suppressive T cells [53]. In an extension to these observations, AMBRA1 is identified as a central regulator of T‐cell homeostasis and cytotoxic potential [18, 54], suggesting AMBRA1 loss in immune cells of the tumor microenvironment may further impair tumor immunity.

We acknowledge some limitations. Gene expression profiling relied on microarray‐based transcriptomic profiling instead of RNA sequencing. Although microarrays offer reliable quantification of annotated genes and enable direct comparison with legacy datasets, they are constrained by probe design, a narrower dynamic range, and limited ability to detect low‐abundance or novel transcripts. As a result, some transcriptional changes may not have been captured. Moreover, the protein–protein interaction work is preliminary and has not been validated with pull down experiments, but the authors feel that this preliminary data may be of interest to the AMBRA1 community.

In summary, our study reveals potential roles of AMBRA1 that can extend beyond autophagy and its role reported in cell proliferation, and it identifies protein interactors of AMBRA1 that can regulate or be regulated through this interaction. Our study suggests that AMBRA1 loss in some melanoma tumors can result in the upregulation of angiogenic and metastatic genes that can be attributed to its role as a tumor suppressor in melanoma.

## Conflict of interest

Authors declare no conflicts of interest.

## Author contributions

MI, JA, and NC conceived and designed the project. MI acquired and curated the data. MI and NC performed formal analysis, validation, investigation, and visualization. MC, IO, and JA contributed to resources and experimental investigation. MI, MC, JA, and NC developed the methodology. MI wrote the original draft of the manuscript. MI, MC, IO, JA, and NC contributed to writing, review, and editing. JA and NC supervised the project and provided project administration.

## Supporting information


**Fig S1.** AMBRA1 mutations do not cluster in a specific domain.
**Fig. S2.** Stain‐free membranes showing total protein loaded.
**Fig. S3.** AMBRA1 levels are restored in shAMBRA1 cell lines after prolonged time of no antibiotic selection.
**Fig. S4.** AMBRA1 regulate hallmark cellular processes on the transcriptional level.
**Fig. S5.** STRING protein analysis color key identifying types of interactions.

**File S1.** DepMap AMBRA1 gene effect (DEMETER2) filtered by Melanoma_RNAi.

**File S2.** DepMap AMBRA1 gene effect (Chronos) filtered by Melanoma_CRISPR.

**File S3.** rAmbra versus rBgal differentially expressed genes.

**File S4.** shAMBRA1 versus shScramble differentially expressed genes.

## Data Availability

Microarray transcriptomic data are available at GEO datasets (GSE175361). Differential gene expression analysis, depmap Chronos scores are included as supporting information tables.

## References

[1] Arnold M , Singh D , Laversanne M , Vignat J , Vaccarella S , Meheus F , Cust AE , de Vries E , Whiteman DC and Bray F (2022) Global burden of cutaneous melanoma in 2020 and projections to 2040. JAMA Dermatol 158, 495–503. 35353115 10.1001/jamadermatol.2022.0160 PMC8968696

[2] Davis LE , Shalin SC and Tackett AJ (2019) Current state of melanoma diagnosis and treatment. Cancer Biol Ther 20, 1366–1379. 31366280 10.1080/15384047.2019.1640032 PMC6804807

[3] Curti BD and Faries MB (2021) Recent advances in the treatment of melanoma. N Engl J Med 384, 2229–2240. 34107182 10.1056/NEJMra2034861

[4] Domingues B , Lopes JM , Soares P and Pópulo H (2018) Melanoma treatment in review. Immunotargets Ther 7, 35–49. 29922629 10.2147/ITT.S134842 PMC5995433

[5] Sood S , Jayachandiran R and Pandey S (2021) Current advancements and novel strategies in the treatment of metastatic melanoma. Integr Cancer Ther 20, 1534735421990078. 33719631 10.1177/1534735421990078 PMC8743966

[6] Fimia GM , Stoykova A , Romagnoli A , Giunta L , Di Bartolomeo S , Nardacci R , Corazzari M , Fuoco C , Ucar A , Schwartz P *et al*. (2007) Ambra1 regulates autophagy and development of the nervous system. Nature 447, 1121–1125. 17589504 10.1038/nature05925

[7] Cianfanelli V , De Zio D , Di Bartolomeo S , Nazio F , Strappazzon F and Cecconi F (2015) Ambra1 at a glance. J Cell Sci 128, 2003–2008. 26034061 10.1242/jcs.168153

[8] Ellis R , Tang D , Nasr B , Greenwood A , McConnell A , Anagnostou ME , Elias M , Verykiou S , Bajwa D , Ewen T *et al*. (2020) Epidermal autophagy and beclin 1 regulator 1 and loricrin: a paradigm shift in the prognostication and stratification of the American joint committee on cancer stage I melanomas. Br J Dermatol 182, 156–165. 31056744 10.1111/bjd.18086 PMC6973157

[9] Ewen T , Husain A , Stefanos N , Barrett P , Jones C , Ness T , Long A , Horswell S , Bosomworth H , Lowenstein J *et al*. (2024) Validation of epidermal AMBRA1 and loricrin (AMBLor) as a prognostic biomarker for nonulcerated American joint committee on cancer stage I/II cutaneous melanoma. Br J Dermatol 190, 549–558. 38006317 10.1093/bjd/ljad459

[10] Di Leo L , Bodemeyer V , Bosisio FM , Claps G , Carretta M , Rizza S , Faienza F , Frias A , Khan S , Bordi M *et al*. (2021) Loss of Ambra1 promotes melanoma growth and invasion. Nat Commun 12, 2550. 33953176 10.1038/s41467-021-22772-2 PMC8100102

[11] Cianfanelli V , Fuoco C , Lorente M , Salazar M , Quondamatteo F , Gherardini PF , De Zio D , Nazio F , Antonioli M , D'Orazio M *et al*. (2015) AMBRA1 links autophagy to cell proliferation and tumorigenesis by promoting c‐Myc dephosphorylation and degradation. Nat Cell Biol 17, 20–30. 25438055 10.1038/ncb3072 PMC4976803

[12] Maiani E , Milletti G , Nazio F , Holdgaard SG , Bartkova J , Rizza S , Cianfanelli V , Lorente M , Simoneschi D , Di Marco M *et al*. (2021) AMBRA1 regulates cyclin D to guard S‐phase entry and genomic integrity. Nature 592, 799–803. 33854232 10.1038/s41586-021-03422-5 PMC8864551

[13] Simoneschi D , Rona G , Zhou N , Jeong YT , Jiang S , Milletti G , Arbini AA , O'Sullivan A , Wang AA , Nithikasem S *et al*. (2021) CRL4(AMBRA1) is a master regulator of D‐type cyclins. Nature 592, 789–793. 33854235 10.1038/s41586-021-03445-y PMC8875297

[14] Sun WL , Wang L , Luo J , Zhu HW and Cai ZW (2018) Ambra1 modulates the sensitivity of breast cancer cells to epirubicin by regulating autophagy via ATG12. Cancer Sci 109, 3129–3138. 30027574 10.1111/cas.13743 PMC6172055

[15] He RQ , Xiong DD , Ma J , Hu XH , Chen G and Sun WL (2018) The Clinicopathological significance and correlative signaling pathways of an autophagy‐related gene, Ambra1, in breast cancer: a study of 25 microarray RNA‐Seq datasets and in‐house gene silencing. Cell Physiol Biochem 51, 1027–1040. 30476925 10.1159/000495483

[16] Ye L , Lin D , Zhang W , Chen S , Zhen Y , Akkouche S , Liang X , Chong CM and Zhong HJ (2024) AMBRA1 drives gastric cancer progression through regulation of tumor plasticity. Front Immunol 15, 1494364. 39720719 10.3389/fimmu.2024.1494364 PMC11666514

[17] Liu J , Chen Z , Guo J , Wang L and Liu X (2019) Ambra1 induces autophagy and desensitizes human prostate cancer cells to cisplatin. Biosci Rep 39, BSR20170770 29101240 10.1042/BSR20170770 PMC6706594

[18] Gottlieb S , Shang W , Ye D , Kubo S , Jiang PD , Shafer S , Xu L , Zheng L , Park AY , Song J *et al*. (2024) AMBRA1 controls the translation of immune‐specific genes in T lymphocytes. Proc Natl Acad Sci USA 121, e2416722121. 39436665 10.1073/pnas.2416722121 PMC11536168

[19] Di Rienzo M , Romagnoli A , Refolo G , Vescovo T , Ciccosanti F , Zuchegna C , Lozzi F , Occhigrossi L , Piacentini M and Fimia GM (2024) Role of AMBRA1 in mitophagy regulation: emerging evidence in aging‐related diseases. Autophagy 20, 2602–2615. 39113560 10.1080/15548627.2024.2389474 PMC11587829

[20] Raskin L , Fullen DR , Giordano TJ , Thomas DG , Frohm ML , Cha KB , Ahn J , Mukherjee B , Johnson TM and Gruber SB (2013) Transcriptome profiling identifies HMGA2 as a biomarker of melanoma progression and prognosis. J Invest Dermatol 133, 2585–2592. 23633021 10.1038/jid.2013.197 PMC4267221

[21] Budden T , Davey RJ , Vilain RE , Ashton KA , Braye SG , Beveridge NJ and Bowden NA (2016) Repair of UVB‐induced DNA damage is reduced in melanoma due to low XPC and global genome repair. Oncotarget 7, 60940–60953. 27487145 10.18632/oncotarget.10902 PMC5308628

[22] Cerami E , Gao J , Dogrusoz U , Gross BE , Sumer SO , Aksoy BA , Jacobsen A , Byrne CJ , Heuer ML , Larsson E *et al*. (2012) The cBio cancer genomics portal: an open platform for exploring multidimensional cancer genomics data. Cancer Discov 2, 401–404. 22588877 10.1158/2159-8290.CD-12-0095 PMC3956037

[23] Tang Z , Kang B , Li C , Chen T and Zhang Z (2019) GEPIA2: an enhanced web server for large‐scale expression profiling and interactive analysis. Nucleic Acids Res 47, W556–w560. 31114875 10.1093/nar/gkz430 PMC6602440

[24] Armstrong JL , Hill DS , McKee CS , Hernandez‐Tiedra S , Lorente M , Lopez‐Valero I , Eleni Anagnostou M , Babatunde F , Corazzari M , Redfern CPF *et al*. (2015) Exploiting cannabinoid‐induced cytotoxic autophagy to drive melanoma cell death. J Invest Dermatol 135, 1629–1637. 25674907 10.1038/jid.2015.45

[25] Ibrahim M , Illa‐Bochaca I , Fa'ak F , Monson KR , Ferguson R , Lyu C , Vega‐Saenz de Miera E , Johannet P , Chou M , Mastroianni J *et al*. (2023) Kinase insert domain receptor Q472H pathogenic germline variant impacts melanoma tumor growth and patient treatment outcomes. Cancers (Basel) 16, 18. 38201446 10.3390/cancers16010018 PMC10778134

[26] Ibrahim M , Illa‐Bochaca I , Jour G , Vega‐Saenz de Miera E , Fracasso J , Ruggles K , Osman I and Schober M (2025) NF1 loss promotes EGFR activation and confers sensitivity to EGFR inhibition in NF1‐mutant melanoma. Cancer Res 85, 3348–3364. 40494652 10.1158/0008-5472.CAN-24-3904 PMC12221223

[27] Gagliardi M , Cotella D , Santoro C , Corà D , Barlev NA , Piacentini M and Corazzari M (2019) Aldo‐keto reductases protect metastatic melanoma from ER stress‐independent ferroptosis. Cell Death Dis 10, 902. 31780644 10.1038/s41419-019-2143-7 PMC6883066

[28] Di Rienzo M , Romagnoli A , Ciccosanti F , Refolo G , Consalvi V , Arena G , Valente EM , Piacentini M and Fimia GM (2022) AMBRA1 regulates mitophagy by interacting with ATAD3A and promoting PINK1 stability. Autophagy 18, 1752–1762. 34798798 10.1080/15548627.2021.1997052 PMC9450973

[29] Pagliarini V , Wirawan E , Romagnoli A , Ciccosanti F , Lisi G , Lippens S , Cecconi F , Fimia GM , Vandenabeele P , Corazzari M *et al*. (2012) Proteolysis of Ambra1 during apoptosis has a role in the inhibition of the autophagic pro‐survival response. Cell Death Differ 19, 1495–1504. 22441670 10.1038/cdd.2012.27 PMC3422474

[30] Maloy A , Alexander S , Andreas A , Nyunoya T and Chandra D (2022) Stain‐free total‐protein normalization enhances the reproducibility of Western blot data. Anal Biochem 654, 114840. 35931182 10.1016/j.ab.2022.114840 PMC10214384

[31] Neris RLS , Dobles AMC and Gomes AV (2021) Western blotting using in‐gel protein labeling as a normalization control: advantages of stain‐free technology. Methods Mol Biol 2261, 443–456. 33421007 10.1007/978-1-0716-1186-9_28 PMC12288770

[32] Aleksander SA , Balhoff J , Carbon S , Cherry JM , Drabkin HJ , Ebert D , Feuermann M , Gaudet P , Harris NL , Hill DP *et al*. (2023) The gene ontology knowledgebase in 2023. Genetics 224, iyad031. 36866529 10.1093/genetics/iyad031 PMC10158837

[33] Ashburner M , Ball CA , Blake JA , Botstein D , Butler H , Cherry JM , Davis AP , Dolinski K , Dwight SS , Eppig JT *et al*. (2000) Gene ontology: tool for the unification of biology. The gene ontology consortium. Nat Genet 25, 25–29. 10802651 10.1038/75556 PMC3037419

[34] Snel B , Lehmann G , Bork P and Huynen MA (2000) STRING: a web‐server to retrieve and display the repeatedly occurring neighbourhood of a gene. Nucleic Acids Res 28, 3442–3444. 10982861 10.1093/nar/28.18.3442 PMC110752

[35] Szklarczyk D , Kirsch R , Koutrouli M , Nastou K , Mehryary F , Hachilif R , Gable AL , Fang T , Doncheva NT , Pyysalo S *et al*. (2023) The STRING database in 2023: protein‐protein association networks and functional enrichment analyses for any sequenced genome of interest. Nucleic Acids Res 51, D638–d646. 36370105 10.1093/nar/gkac1000 PMC9825434

[36] Nazio F , Po A , Abballe L , Ballabio C , Diomedi Camassei F , Bordi M , Camera A , Caruso S , Caruana I , Pezzullo M *et al*. (2021) Targeting cancer stem cells in medulloblastoma by inhibiting AMBRA1 dual function in autophagy and STAT3 signalling. Acta Neuropathol 142, 537–564. 34302498 10.1007/s00401-021-02347-7 PMC8357694

[37] Arafeh R , Shibue T , Dempster JM , Hahn WC and Vazquez F (2025) The present and future of the cancer dependency map. Nat Rev Cancer 25, 59–73. 39468210 10.1038/s41568-024-00763-x

[38] Huttlin EL , Bruckner RJ , Navarrete‐Perea J , Cannon JR , Baltier K , Gebreab F , Gygi MP , Thornock A , Zarraga G , Tam S *et al*. (2021) Dual proteome‐scale networks reveal cell‐specific remodeling of the human interactome. Cell 184, 3022–3040. 33961781 10.1016/j.cell.2021.04.011 PMC8165030

[39] Mathiassen SG , De Zio D and Cecconi F (2017) Autophagy and the cell cycle: a complex landscape. Front Oncol 7, 51. 28409123 10.3389/fonc.2017.00051 PMC5374984

[40] Sun WL (2016) Ambra1 in autophagy and apoptosis: implications for cell survival and chemotherapy resistance. Oncol Lett 12, 367–374. 27347152 10.3892/ol.2016.4644 PMC4906955

[41] Chaikovsky AC , Li C , Jeng EE , Loebell S , Lee MC , Murray CW , Cheng R , Demeter J , Swaney DL , Chen S‐H *et al*. (2021) The AMBRA1 E3 ligase adaptor regulates the stability of cyclin D. Nature 592, 794–798. 33854239 10.1038/s41586-021-03474-7 PMC8246597

[42] Akatsuka H , Kashikawa T , Masuhara K , Tokusanai M , Li C , Iida Y , Okada‐Yamaguchi C , Okada Y , Tanaka M , Suzuki T *et al*. (2025) Molecular and genetic evidence for the role of AMBRA1 in suppressing S‐phase entry and tumorigenesis. iScience 28, 113054. 40734673 10.1016/j.isci.2025.113054 PMC12304916

[43] Atzori MG , Ceci C , Ruffini F , Scimeca M , Cicconi R , Mattei M , Lacal PM and Graziani G (2022) The anti‐vascular endothelial growth factor receptor 1 (VEGFR‐1) D16F7 monoclonal antibody inhibits melanoma adhesion to soluble VEGFR‐1 and tissue invasion in response to placenta growth factor. Cancers (Basel) 14, 5578. 36428669 10.3390/cancers14225578 PMC9688925

[44] Metzner T , Bedeir A , Held G , Peter‐Vörösmarty B , Ghassemi S , Heinzle C , Spiegl‐Kreinecker S , Marian B , Holzmann K , Grasl‐Kraupp B *et al*. (2011) Fibroblast growth factor receptors as therapeutic targets in human melanoma: synergism with BRAF inhibition. J Invest Dermatol 131, 2087–2095. 21753785 10.1038/jid.2011.177 PMC3383623

[45] Wang H , Lee S , Nigro CL , Lattanzio L , Merlano M , Monteverde M , Matin R , Purdie K , Mladkova N , Bergamaschi D *et al*. (2012) NT5E (CD73) is epigenetically regulated in malignant melanoma and associated with metastatic site specificity. Br J Cancer 106, 1446–1452. 22454080 10.1038/bjc.2012.95 PMC3326678

[46] Ceci C , Ruffini F , Falconi M , Atzori MG , Falzon A , Lozzi F , Iacovelli F , D'Atri S , Graziani G and Lacal PM (2024) Pharmacological inhibition of PDGF‐C/neuropilin‐1 interaction: a novel strategy to reduce melanoma metastatic potential. Biomed Pharmacother 176, 116766. 38788599 10.1016/j.biopha.2024.116766

[47] Kodama S , Podyma‐Inoue K , Uchihashi T , Kurioka K , Takahashi H , Sugauchi A , Takahashi K , Inubushi T , Kogo M , Tanaka S *et al*. (2021) Progression of melanoma is suppressed by targeting all transforming growth factor‐β isoforms with an fc chimeric receptor. Oncol Rep 46, 1. 10.3892/or.2021.8148 PMC8317165 34296292

[48] Kaminaka R , Takahashi N , Nishizawa M , Sasaki S , Sugihara Y , Matsumoto M , Taniguchi R , Zhou Y , Hayakawa Y , Sakurai H *et al*. (2025) SOX10 regulates melanoma metastasis through the IRF1‐ITGA3/EphA2‐FAK pathway. Cancer Sci 116, 3052–3065. 40808260 10.1111/cas.70173 PMC12580875

[49] Bahar ME , Kim HJ and Kim DR (2023) Targeting the RAS/RAF/MAPK pathway for cancer therapy: from mechanism to clinical studies. Signal Transduct Target Ther 8, 455. 38105263 10.1038/s41392-023-01705-z PMC10725898

[50] Hu Q , Tanasa B , Trabucchi M , Li W , Zhang J , Ohgi KA , Rose DW , Glass CK and Rosenfeld MG (2012) DICER‐and AGO3‐dependent generation of retinoic acid‐induced DR2 Alu RNAs regulates human stem cell proliferation. Nat Struct Mol Biol 19, 1168–1175. 23064648 10.1038/nsmb.2400 PMC3743530

[51] Kloth K , Lozic B , Tagoe J , Hoffer MJV , Van der Ven A , Thiele H , Altmüller J , Kubisch C , Au PYB , Denecke J *et al*. (2021) ANK3 related neurodevelopmental disorders: expanding the spectrum of heterozygous loss‐of‐function variants. Neurogenetics 22, 263–269. 34218362 10.1007/s10048-021-00655-4 PMC8426245

[52] Zhu S , Zhang X , Liu W , Zhou Z , Xiong S , Chen X and Peng C (2025) Vinburnine potentiates anti‐PD1 immunotherapy in melanoma through IL‐24 secretion via P38/MAPK/ATF3 signaling. J Exp Clin Cancer Res 44, 255. 40866941 10.1186/s13046-025-03521-5 PMC12382293

[53] Frias A , Di Leo L , Antoranz A , Nazerai L , Carretta M , Bodemeyer V , Pagliuca C , Dahl C , Claps G , Mandelli GE *et al*. (2023) Ambra1 modulates the tumor immune microenvironment and response to PD‐1 blockade in melanoma. J Immunother Cancer 11, e006389. 36868570 10.1136/jitc-2022-006389 PMC9990656

[54] Migliore L , Cianfanelli V , Zevolini F , Gesualdo M , Marzuoli L , Patrussi L , Ulivieri C , Marotta G , Cecconi F , Finetti F *et al*. (2024) An AMBRA1, ULK1 and PP2A regulatory network regulates cytotoxic T cell differentiation via TFEB activation. Sci Rep 14, 31838. 39738384 10.1038/s41598-024-82957-9 PMC11685475

