# Supplementary material for: Loss of AMBRA1 activates MAPK and angiogenesis signaling pathways in melanoma cells
Source: FEBS Open Bio. 2026 Jun 2:10.1002/2211-5463.70281. Online ahead of print. doi: 10.1002/2211-5463.70281 (PMC13398474; doi:10.1002/2211-5463.70281)
Supplement: Supplementary file 1 — Fig S1. AMBRA1 mutations do not cluster in a specific domain. Fig. S2. Stain‐free membranes showing total protein loaded. Fig. S3. AMBRA1 levels are restored in shAMBRA1 cell lines after prolonged time of no antibiotic selection. Fig. S4. AMBRA1 regulate hallmark cellular processes on the transcriptional level. Fig. S5. STRING protein analysis color key identifying types of interactions. [file FEB4-9999-0-s002.docx]

**
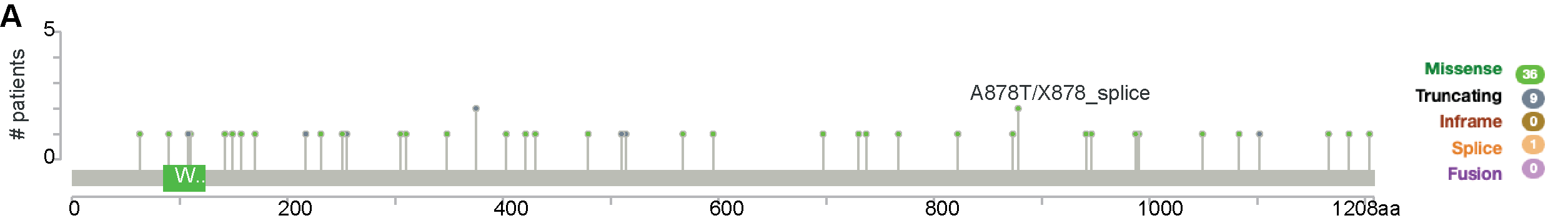
**

**Fig. S1 AMBRA1 mutations do not cluster in a specific domain.** (**A**) Lollipop plot showing AMBRA1 mutations in SKCM pan cancer study. Each mutation is represented by a lollipop on AMBRA1 gene.


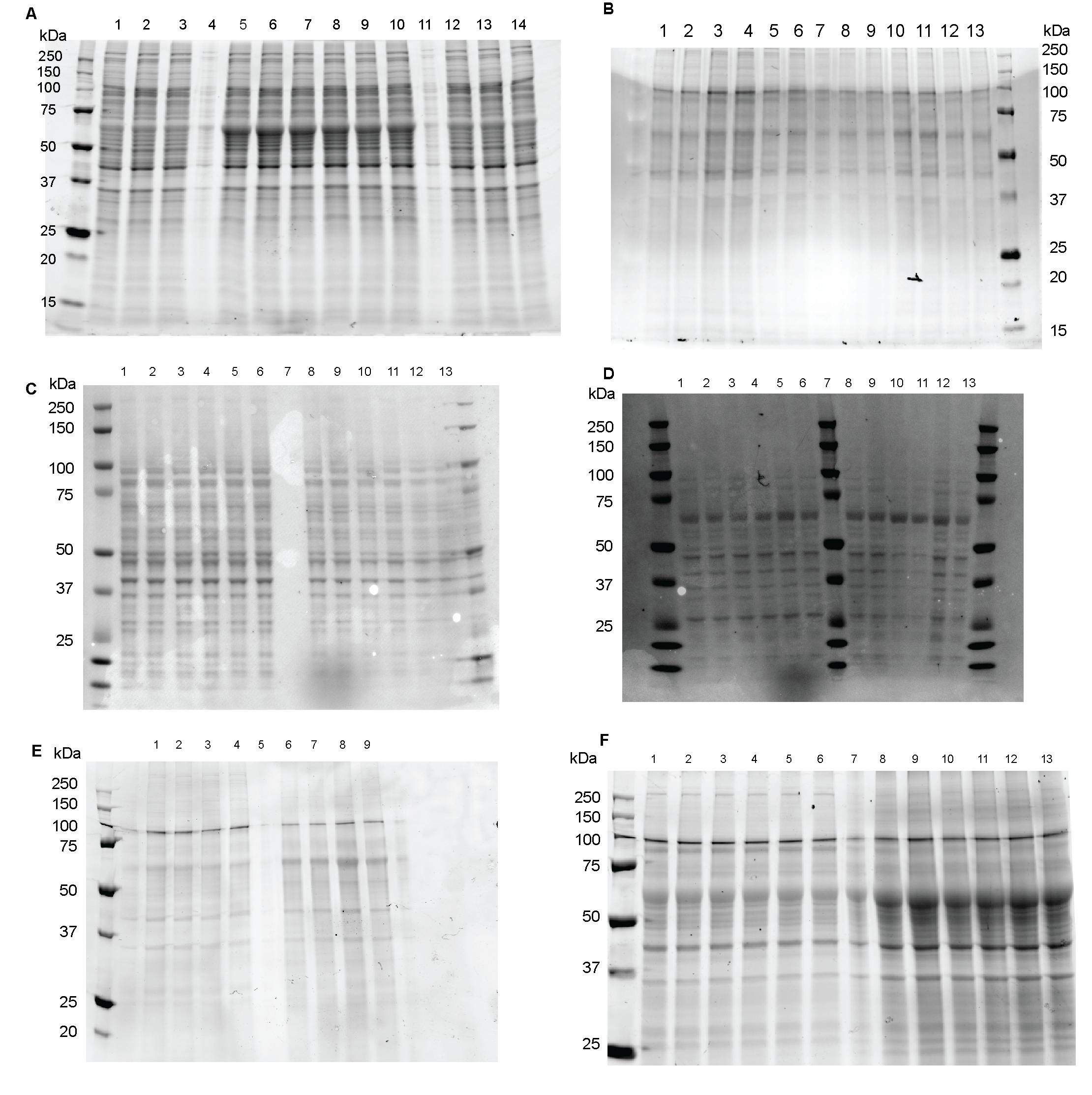


**Fig. S2 Stain free membranes showing total protein loaded of** (**A**) rAMBRA overexpression (Fig. 2A), lanes 1-3 and 5-7 are three technical of two biological rAMBRA1 replicates and lanes 8-10 and 12-14 are three technical of two biological rBgal replicates **(B)** AMBRA1 knockdown in A375 (Fig. 2B), lanes 1-6 are two technical of three biological shScramble replicates and lanes 8-13 are two technical of three biological shAMBRA1 replicates. **(C)** AMBRA1 knockdown (Fig. 2H), lanes 1-6 are two technical of three biological shScramble replicates and lanes 8-13 are two technical of three biological shAMBRA1 replicates. **(D)** AMBRA1 knockdown (Fig. 2J), lanes 1-6 are two technical of three biological shScramble replicates and lanes 8-13 are two technical of three biological shAMBRA1 replicates. **(E)** FLT1 overexpression in shAMBRA1 (Fig. 3D), lanes 1 to 4 are two technical replicates of two shAMBRA1 biological replicates. Lanes 6 to 9 are two technical replicates of two shScramble biological replicates. **(F)** WNT5A overexpression in shAMBRA1 (Fig. 3D), lanes 1 to 6 are two technical replicates of three shAMBRA1 biological replicates. Lanes 6 to 9 are two technical replicates of three shScramble biological replicates.

**
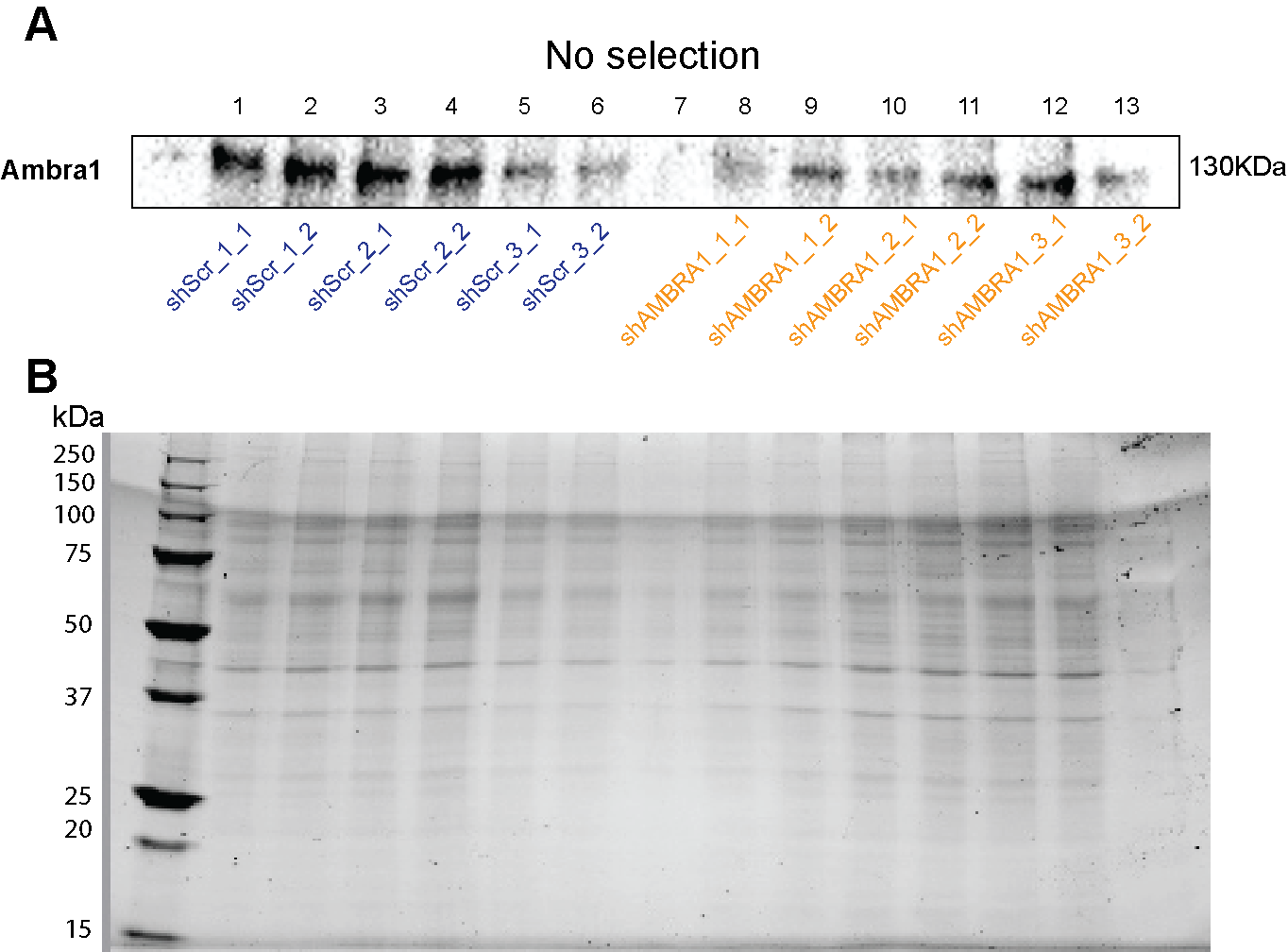
**

**Fig. S3 AMBRA1 levels are restored in shAMBRA1 cell lines after prolonged time of no antibiotic selection.** (**A**) Western blot analysis of AMBRA1 levels comparing shScramble to shAMBRA1 after two weeks of removing antibiotic selection. Lanes 1 to 6 are two technical replicates of three shScramble biological replicates. Lanes 8 to 13 are two technical replicates of three shAMBRA1 biological replicates. **(B)** Stain free membrane showing protein loading controls of (A).


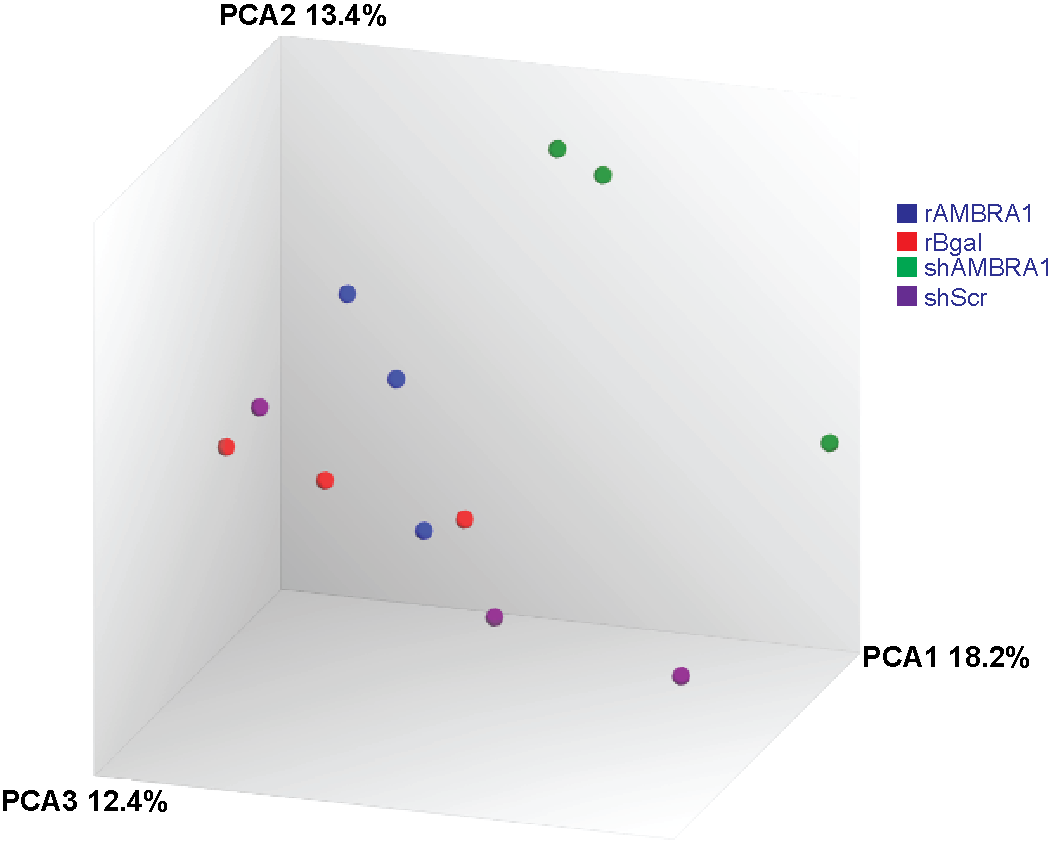


**Fig. S4 AMBRA1 regulate hallmark cellular processes on the transcriptional level** (**A**) PCA plot of cells overexpressing AMBRA1 (rAMBRA), overexpression control (rBgal), AMBRA1 knockdown (shAMBRA1), and knockdown control (shScramble).
